# New born screening program in India: ICMR multicentric experience

**DOI:** 10.1186/1755-8166-7-S1-I40

**Published:** 2014-01-21

**Authors:** Roli Mathur

**Affiliations:** 1 Indian Council of Medical Research, New Delhi – 110029, India

## 

Inborn Metabolic Disorders (IMD) are common genetic disorders imposing huge burden on health care infrastructure though many of these are treatable conditions. Indian Council of Medical Research (ICMR) recognized the need of initiating a multicentre Task Force (NTF-IMD) study to systematically collect data for congenital hypothyroidism and congenital adrenal hyperplasia to help in early diagnosis and management to prevent disability.

This study was done to evaluate the feasibility of a newborn screening for different geo-ethnic regions of the Indian Population and attempt to define the incidence of the selected inborn metabolic errors i.e., CH and CAH in the population and also to develop the capability of treating and diagnosing inborn errors of metabolism.

A series of brainstorming sessions were conducted between 2008-2013 at 5 regional centers in the country before the start of the study. The NTF-IMD Group developed a common protocol for implementation at all study sites for comprehensive screening, management, treatment of affected newborns, counseling, high risk screening in sick newborns, enrolment in quality assurance program, development of tools for advocacy, setting up of a dedicated website etc. A sample size of 100,000 newborns was set as a target and 1000 sick children admitted in ICU’s at study sites were also included.

Coordinated effort of a dedicated team of investigators at 5 newborn screening centers, 3 high risk screening centers, data coordination centre, quality assurance centre and central coordination centers could lead to successful completion of newborn in more than 1,00,000 newborns from both urban and rural areas. The study involved the use of common protocols and involved data compilation and analysis to prepare comprehensive results from all centers and set a data representative for the country following thorough quality assurance procedures. The study helped to establish the incidence of CH and CAH in Indian population and prevented disability in affected newborns following early diagnosis and treatment. The study has far reaching implications as it helped to build regional capacity and in setting up an invaluable model for future newborn screening programs in India as well as other developing countries.

